# Diagnostic Errors: Association with level of expertise, effect of time taken to reach the diagnosis and utilization of differential diagnosis checklists among postgraduate trainees

**DOI:** 10.12669/pjms.38.8.5392

**Published:** 2022

**Authors:** Hasham Khan, Usman Mahboob, Tariq Ahmad

**Affiliations:** 1 Dr. Hasham Khan, BDS; M. Sc (University of London); MHPE (KMU). Professor of Paediatric Dentistry, Khyber College of Dentistry (KCD), Peshawar, Pakistan; 2 Dr. Usman Mahboob, MBBS; MPH; FHEA; DHPE; Fellow FAIMER. Associate Professor, Institute of Health Professions Education & Research (IHPER) Khyber Medical University (KMU), Peshawar, Pakistan; 3 Dr. Tariq Ahmad, BDS; FCPS. Assistant Professor Oral & Maxillofacial Surgery, Khyber College of Dentistry (KCD), Peshawar, Pakistan

**Keywords:** Diagnostic Errors, Expertise, Training Level, Time Taken to Reach Diagnosis, Differential Diagnosis Checklists, Maxillofacial Surgery Trainees

## Abstract

**Objectives:**

The objectives of this study were to evaluate: (1) The association between level of training (expertise) and rate of diagnostic errors. (2) The effect of time taken to reach a diagnosis on the frequency of diagnostic errors. (3) The effect of utilization of differential diagnosis checklists in reducing the frequency of diagnostic errors.

**Methods:**

The study was carried out from November 2020 till April 2021 in Peshawar. The participants included FCPS Part-II trainees of Maxillofacial Surgery undergoing training in five centres. Thirty written case scenarios were prepared and validated, ten scenarios for each of the three objectives. To evaluate the association between training level (expertise) and the rate of diagnostic errors, two groups of trainees (1^st^ year group and 4^th^ year group) were formed and given ten same case scenarios for diagnosis. To evaluate the effect of time taken to reach diagnosis on the frequency of diagnostic errors, two groups of 4^th^ year trainees (fast group and slow group) were formed by random allocation of participants to groups and given ten similar case scenarios for diagnosis. Fast group was given 15-minutes whereas slow group was given 30-minutes to respond. To evaluate the effect of utilization of differential diagnosis checklists in reducing diagnostic errors, again two groups of 4^th^ year trainees were formed by random allocation of participants to groups and given ten similar case scenarios for diagnosis. One group was given differential diagnosis checklists for the scenarios and the other none.

**Results:**

In this study, participants included were 1^st^ year (n=36) and 4^th^ year (n=36) trainees of Maxillofacial Surgery. The results showed that training level or expertise was significantly associated with the rate of diagnostic errors (*p* = 0.002). Time taken to reach diagnosis and differential diagnosis checklists have no significant effect on the frequency of diagnostic errors (*p* = 0.74 and 0.56 respectively).

**Conclusions:**

Training level (expertise) has significant effect on the frequency of diagnostic errors whereas no significant effect was recorded for time (time taken to reach diagnosis) and differential diagnosis checklists on the rate of diagnostic errors.

## INTRODUCTION

In medicine, diagnostic errors are common and costly, and result in almost 70% of medical errors.^1^ Cognitive diagnostic errors (cognitive biases or failed heuristics) are difficult to understand and prevent as compared to health care system errors (lack of communication and co-ordination among health care professionals) which are easily identifiable and actionable.^2^,^3^

Diagnostic process (clinical reasoning) takes place through a dual process model of clinical reasoning, that is; an intuitive, rapid, automatic and pattern-based decision-making called system-1 and an analytical, effortful, logical reasoning called system-2.^4^ There is a prevalent view in medical literature that diagnostic errors primarily originate in System-1 (fast thinking) and are subjected to correction by System-2 (slow thinking).^5^

There is some controversy as to the etiology of diagnostic errors especially the knowledge deficit (expertise).^6^-^8^ Controversy also exists as to the effect of time pressure (workload imbalance) on the frequency of diagnostic errors.^5^,^6^,^9^ Checklists have been introduced to reduce diagnostic errors but not much tested.^10^

To reduce or eliminate diagnostic errors, we need to understand the factors contributing to diagnostic errors, especially the level of training (expertise), and time taken to reach diagnosis, and the role of differential diagnosis checklists, in our local context.

The objectives of this study were therefore to evaluate: (1) The association between level of training (expertise) and rate of diagnostic errors. (2) The effect of time taken in reaching the diagnosis on the frequency of diagnostic errors. (3) The effect of differential diagnosis checklists in reducing the frequency of diagnostic errors.

## METHODS

The study was carried out from November 2020 till April 2021 in Peshawar. The participants included FCPS (Fellowship of College of Physicians & Surgeons Pakistan) Part-II trainees of Oral & Maxillofacial Surgery (OMFS) undergoing training in five training centres in Peshawar, namely: Khyber College of Dentistry (KCD), Hayatabad Medical Complex (HMC), Sardar Begum Dental College (SBDC), Combined Military Hospital (CMH) and Rehman College of Dentistry (RCD). The FCPS is a four-year clinical training program. A census sample was taken due to the limited number of 4^th^ year trainees of OMFS at all of the above centres.

The study design was dual, Causal-Comparative for determination of any association between training level (expertise) and the rate of diagnostic errors and; - experimental (Posttest-Only Design) to evaluate the effect of time taken to reach a diagnosis and the utilization of differential diagnosis checklists on the frequency of diagnostic errors (Fig-I). Ethical approval was obtained from the Khyber Medical University Ethics Board (Reference No. Dir/KMU-EB/DE/000791). This study utilized written case scenarios for the diagnoses as it was not prudent to conduct this study on actual patients. The protocols and instruments were piloted and adjusted for the main study.

**Fig.1:**
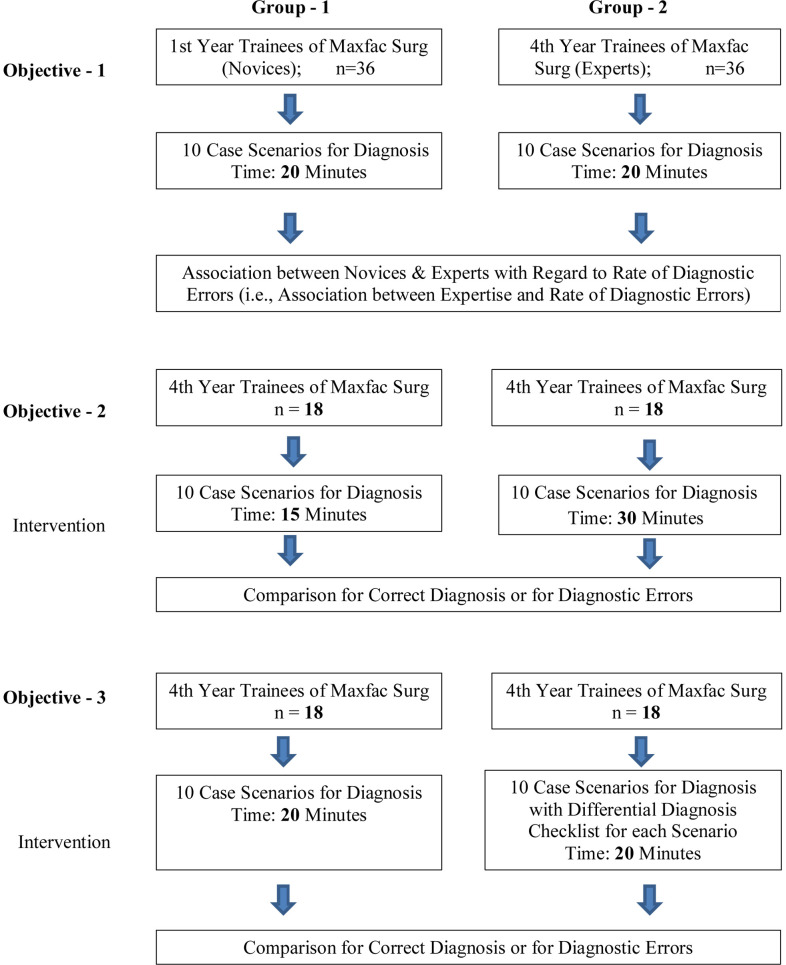
Flow Chart of the study design on Diagnostic Errors.

For each objective, ten written case scenarios were used for establishing the diagnoses by the participants (in total 30 written case scenarios were prepared for the three objectives). Differential diagnosis checklists were prepared for each of the ten scenarios used to evaluate the role of differential diagnosis checklists in reducing diagnostic errors. All the 30 scenarios were standardized through review and feedback from five consultant maxillofacial surgeons - each of them having more than ten years of postgraduate teaching experience.

To evaluate the association between training level (expertise) and the rate of diagnostic errors, two groups of trainees, 1st-year group and 4th-year group, were formed according to their level of training. Both the groups were given ten same written case scenarios for diagnosis. Twenty minutes were given to both groups for a response. To evaluate the effect of time taken to reach a diagnosis on the frequency of diagnostic errors, two groups of trainees were formed but of the same training level, that is, 4th year by random allocation of the participants to groups. Allocations of male & female trainees were done separately to get an equal number of trainees (gender-wise) in both groups. Both the groups were given ten same written case scenarios for diagnosis. One group was given 15-minutes (fast group) and the other 30-minutes (slow group) to answer all the ten scenarios, to see the effect of time on the frequency of diagnostic errors. To evaluate the role of differential diagnosis checklists in reducing the rate of diagnostic errors**,** again two groups of trainees were formed (same training level, 4th year) by random allocation of the participants to groups. Allocations of male & female trainees were again done separately to get an equal number of trainees (gender-wise) in both groups. Both groups were given ten same written case scenarios for diagnosis. The time for response was fixed at 20-minutes. One group was given the checklists of differential diagnoses for each written scenario and the other none.

Those trainees who had joined the maxillofacial unit in the last three months were excluded from the study as they were not having any experience or knowledge of the speciality for appropriate diagnosis and decision-making. The data were entered in a computer and analyzed using SPSS Version # 22.

## RESULTS

The census sample consisted of 1^st^ year (n=36) and 4^th^ year (n=36) FCPS trainees of Oral & Maxillofacial Surgery (Table-I). Kolmogorov-Smirnov and Shapiro-Wilk test analysis showed that the data were not normally distributed. Hence, Mann-Whitney U test was used for all the three variables to determine any significant difference in the mean number of the diagnostic errors between the groups.

**Table-I:** Descriptive Statistics for the three objectives of the study.

Association of Experience Level with Rate of Diagnostic Errors		
Variable	Groups	Frequency (Number of participants)
Level of Experience	1^st^ Year PG Trainees*	36
	4th Year PG Trainees*	36
***Effect of Time (Allocated for diagnosis) on Rate of Diagnostic Errors***		
Effect of Time	Fast Group (Allocated 15 minutes)	18
	Slow Group (Allocated 30 minutes)	18
***Role of Differential Diagnosis Checklists in Reducing Diagnostic Errors***		
Role of Checklists	With Checklists	18
	Without Checklists	18
***Mean and Standard Deviation***		
***Errors Score for Variables***	***Mean***	***S D***
Errors Score (Association with Expertise); n = 72	2.82	1.938
Errors Score (Effect of Time); n = 36	3.06	1.897
Errors Score (Role of Checklists); n = 36	3.53	1.765

* Post Graduate Trainees.

### Association between Level of Training (Expertise) and the Rate of Diagnostic Errors:

There was a significant (p=0.002) difference in the mean number of the diagnostics errors between the 1^st^ year trainees’ group and 4th year trainees’ group (Table-II). Logistic regression was used to calculate the Odds Ratio to measure the association. The value of the Odds Ratio was 0.64 (less than 1.00) which meant that as compared to 1st year, the 4th year trainees were 0.64 times less likely to commit diagnostic errors (Table-II).

**Table-II:** Group comparisons for the objectives of the study.

Objective 1: Association of Level of Experience with Rate of Diagnostic Errors Mean Error Score Difference Between 1st Year and 4^th^ Year PG Trainees (Total n=72)					

Variable	Type of groups	N	Mean Score	Sum of Scores	p value*
Expertise	1^st^ Year PG Trainees**	36	44.18	1590.50	0.002
	4^th^ year PG Trainees**	36	28.82	1037.50	

***Odds Ratio for Association of Level of Experience with the Rate of Diagnostic Errors***					

Odds Ratio - Exp (B)	P value		95% Confidence Interval		

0.64	0.002		Lower	Upper	
			0.48	0.85	

*Dependent variable (Dichotomous) = 1st Year and 4^th^ Year PG Trainees Independent variable (Covariate) = Error score ***Objective 2: Effect of Time (Allocated for the Diagnosis) on Rate of Diagnostic Errors*** Mean Error Score Difference Between Fast and Slow Groups (Total n=36)*					

Variable	Type of groups	N	Mean Score	Sum of Scores	*p*-value*

Effect of Time on Diagnostic Error Score	Fast Group (Allocated 15 minutes)	18	17.94	323.00	0.74
	Slow Group (Allocated 30 minutes)	18	19.06	343.00	

****Objective 3: Effect of Using Differential Diagnosis Checklists on Diagnostic Errors***Mean Error Score Difference Between Checklist Group and Without Checklist Group (Total n=36)*					

Variable	Type of groups	N	Mean Score	Sum of Scores	P-value*

Effect of Checklists on	Group with Checklists	18	17.50	315.00	0.56
Diagnostic Error Score	Group without Checklists	18	19.50	351.00	

*p* value <0.05 taken as significant,

* Mann Whitney U test,

** Post Graduate Trainees.

### Effect of Time (time taken to reach the diagnosis) on the Rate of Diagnostic Errors:

There was no significant (p=0.74) difference between the mean diagnostic error scores in the fast group (allocated 15 minutes) and the slow group (allocated 30 minutes) for the diagnosis (Table-II). We found that there is a trend of more diagnostic errors on part of the slow group. The fast group took an average of 52 seconds per case (written scenario for diagnosis) as compared to 68 seconds for the slow group (p = .001).

### Use of Differential Diagnosis Checklists in Reducing the Diagnostic Errors:

Here also, there was no significant (p=0.56) difference in the mean diagnostic error scores between the group that utilized the differential diagnosis checklists and those who did not (Table-II). However, there was a trend of more diagnostic errors on part of the study group without checklists.

## DISCUSSION

The results of this study revealed that training level of the Part-II FCPS Trainees in OMFS was significantly associated with the rate of diagnostic errors. Literature also shows that with increasing expertise (and knowledge), the chance of diagnostic errors decreases.^6^,^11^ Some other studies on postgraduate trainees have also provided evidence that senior trainees (residents) have lower diagnostic error rates than junior trainees.^12^-^14^ In a retrospective chart review study by Zwaan and colleagues found that insufficient knowledge was the basis for clinical errors.^15^ As far as the strength of association between diagnostic errors and expertise is concerned, previous studies have shown moderate to strong positive correlation.^9^,^16^

A few studies are not in agreement in this aspect of our study. A clinician possessing adaptive expertise and basic knowledge (with any experience level) may make the correct diagnosis in simple as well as unfamiliar and complex cases using his reflective, logical and clinical reasoning skills.^7^ A study carried out by Mamede et al. revealed that greater expertise (experience) without conscious thinking may not reduce diagnostic errors in complex cases.^17^

In our study, the significant association found between the level of experience and diagnostic errors could be attributed to the experiential knowledge and analytical knowledge to some extent, because it was observed that the response time was shorter for most of the 4^th^ year trainees when compared to the 1^st^ year. This observation gives the impression of system – 1 processing.

The results of our study also revealed that time allocated to reach the diagnosis had no significant bearing on the rate of diagnostic errors. These findings are in agreement with those of a previous study in which participants were tested for accuracy in diagnosis on written case scenarios associated with shorter or longer times to diagnoses.^9^ Similarly a study by Norman et al revealed that simply encouraging diagnosticians to slow down is not sufficient to increase diagnostic accuracy or time taken to diagnosis does not affect the frequency of diagnostic errors.^5^ However, one recent study showed that severe constraint of time does increase diagnostic errors by novices.^18^

On the contrary, some studies have found that taking more time to diagnose (slowing down) and reflecting on one’s clinical decisions lead to more accurate diagnoses.^17^,^19^ Mamede and colleagues reported a weak positive relationship between time (time taken to reach diagnosis) and accuracy.^20^ Another study suggested that diagnostic errors may be a consequence of incomplete information and, hence, shorter times to reach a diagnosis.^8^

Most diagnostic errors are made due to our failure to take into consideration the correct diagnosis^8^,^21^ and this problem may be avoided by using a set of differential diagnosis checklists.^10^

The results of our investigation revealed that diagnostic checklists have no significant effect on the rate of diagnostic errors though more diagnostic errors were seen in the group not using checklists. Very few studies have tested checklists in practice. The results of our study are in agreement with an investigation carried out by Ely and Graber.^22^ Similarly, another study carried out on ECG interpretation revealed that checklists did not improve diagnostic performance significantly.^23^

On contrary, studies conducted on medical students found that the use of differential diagnosis checklists improved diagnostic performance.^24^,^25^ A study by Sibbald et al. and his colleagues found that the checklists were helpful to clinicians regardless of expertise level, especially to novices.^26^ The statistically insignificant findings of our study regarding the role of differential diagnosis checklists in reducing diagnostic errors may be due to the small sample size. It is suggested that these may be tested in studies with large sample sizes.

### Limitations of the study:

This study had a small sample size which might have affected results though a census sample was taken. This study was designed to be conducted on postgraduate trainees of a dental specialty and maxillofacial surgery has the maximum number of trainees in the postgraduate centres at Peshawar. Another limitation was that the maxillofacial surgery trainee was considered to be a 4th year trainee without taking into consideration whether he had just started 4th year or was at the end of 4th year of his training. This might have affected the results taking into consideration the difference in experience which a 4th year trainee might have at the start and the end of the year.

## CONCLUSIONS

Training level (expertise) was significantly associated with the rate of diagnostic errors, with senior trainees less likely to commit diagnostic errors as compared to the novices. Time allocated for the diagnosis had no significant effect on the rate of diagnostic errors. In fact, a trend of more diagnostic errors was seen on part of the trainees taking more time to diagnoses (slow group).Use of differential diagnosis checklists had no significant effect on the rate of diagnostic errors, though a trend for more diagnostic errors was seen on part of the group without checklists.

### Authors Contribution:

**HK:** Development of idea and study design, data collection, statistical analysis and manuscript writing.

**UM:** Idea generation, development of study design, review and final approval of manuscript.

**TA:** Helped in development of scenarios and data collection at KCD.

All authors are in agreement to be accountable for all aspects of the work in ensuring that questions related to the accuracy or integrity of any part of the work are appropriately investigated and resolved.
